# Canada's implementation of the Paragraph 6 Decision: is it sustainable public policy?

**DOI:** 10.1186/1744-8603-3-12

**Published:** 2007-12-06

**Authors:** Jillian C Cohen-Kohler, Laura C Esmail, Andre Perez Cosio

**Affiliations:** 1 Leslie Dan Faculty of Pharmacy, University of Toronto, Toronto, Canada 144 College Street, Toronto, ON M5S 3M2, Canada

## Abstract

**Background:**

Following the Implementation of Paragraph 6 of the Doha Declaration on TRIPS and Public Health, Canada was among the first countries globally to amend its patent law, which resulted in Canada's Access to Medicines Regime (CAMR). CAMR allows the production and export of generic drugs to developing countries without the requisite manufacturing capacity to undertake a domestic compulsory license. CAMR has been the subject of much criticism lodged at its inability to ensure fast access to urgent medicines for least developing and developing countries in need. Only recently did the Canadian government grant Apotex the compulsory licenses required under CAMR to produce and export antiretroviral therapy to Rwanda's population.

**Methods:**

The objective of this research is to investigate whether the CAMR can feasibly achieve its humanitarian objectives given the political interests embedded in the crafting of the legislation. We used a political economy framework to analyze the effect of varied institutions, political processes, and economic interests on public policy outcomes. In-depth, semi-structured interviews were conducted with nineteen key stakeholders from government, civil society and industry. Qualitative data analysis was performed using open-coding for themes, analyzing by stakeholder group.

**Results:**

CAMR is removed from the realities of developing countries and the pharmaceutical market. The legislation needs to include commercial incentives to galvanize the generic drug industry to make use of this legislation. CAMR assumes that developing country governments have the requisite knowledge and human resource capacity to make use of the regime, which is not the case. The legislation does not offer sufficient incentives for countries to turn to Canada when needed drugs may be procured cheaply from countries such as India. In the long term, developing and least developing countries seek sustainable solutions to meet the health needs of their population, including developing their own capacity and local industries.

**Conclusion:**

CAMR is symbolically meaningful but in practice, limited. The Rwanda case will be noteworthy in terms of the future of the legislation. To meet its intended international health objectives, this legislation needs to be better informed of developing country needs and global pharmaceutical market imperatives. Finally, we contend that serious public policy change cannot strike a balance between all vested interests. Above all, any feasible policy that aims to facilitate compulsory licensing must prioritize public health over trade or economic interests.

## Background

Following the Implementation of Paragraph 6 of the Doha Declaration on TRIPS and Public Health [1], Canada was among the first countries globally to amend its patent law and as such created Canada's Access to Medicines Regime (CAMR) [2]. Passed in 2004, CAMR allows the production and export of generic drugs to developing countries without the requisite pharmaceutical manufacturing capacity to undertake a domestic compulsory license. Despite its worthwhile humanitarian goals, CAMR is fraught with deficiencies, as critics elsewhere have discussed [3,4]. These include a complicated application process for countries to undertake, a limited list of eligible medicines, restrictions on NGOs as eligible importers, requirements to declare a national emergency and restrictions that prevent re-exportation to facilitate bulk procurement. Some argue that these restrictions go above and beyond what is required by the WTO Paragraph 6 Decision [4].

Since its implementation, no pharmaceuticals have been exported to a country in need, although recent events suggest this situation may change quickly. In July 2007, Rwanda became the first country to attempt to use the regime by notifying the World Trade Organization of its intention to import a shipment of a triple fixed-dose antiretroviral therapy from Apotex, a Canadian generic drug manufacturer [5]. On September 19, the Canadian patent commissioner granted Apotex a compulsory license to produce and export this drug, which is held under patents by three different companies, GlaxoSmithKline (GSK), Shire, and Boehringer Ingelheim [6]. If successful, these efforts will result in the delivery of 260,000 packs of antiretroviral therapy to Rwanda, which would be enough to treat 21,000 patients for one year [6]. Although it is still not clear how the drug will be distributed, this represents some promise for legislation and it would be watched closely by health policy makers and activists given it will be the first test case of CAMR and the WTO Paragraph 6 Decision.

Global drug access research is multidisciplinary in scope, but there is a growing interest in how political economy can have an impact on drug access. Much of this literature focuses on the international politics of the TRIPS negotiations and bilateral trade negotiations, and their impact on public health outcomes. For example, F.M. Abbott analyzed the WTO negotiation strategies and policy context which led to the current shape of the Paragraph 6 Decision [4]. His analysis illustrates the effects of coercive economic and political power on the outcome of these negotiations. More recently, Kerry and Lee analyzed global policy debates on TRIPS and bilateral trade agreements. They conclude that despite affirmation of the Doha Declaration and the Paragraph 6 Decision in principle, their implementation is dramatically hindered by inadequate capacity in low to middle income countries and pressure from powerful trading partners to avoid using such measures [7]. Our research on CAMR aims to contribute this emerging literature by unpacking the lack of stakeholder consensus on this issue, to identify where the incongruence between the interest groups lie, and to inform pharmaceutical public policy.

### Research objectives

The objective of our research was to examine the perceptions and attitudes of CAMR's potential beneficiaries and users towards CAMR. While the recent request by Rwanda to Canada suggests that the legislation is operational, our research aims to explore the potential of CAMR to reach its objectives consistently and with global impact. We pursue this by investigating what incentives it provides to engage the cooperation of the private sector, what potential barriers and opportunities exist for developing countries to apply compulsory licensing, and by examining how well CAMR serves developing countries' needs of access to affordable medicines. Based on our findings, we draw conclusions about how well CAMR may achieve its objective as a legislation providing humanitarian aid – to facilitate access to essential medicines in least developing and developing countries with insufficient or no manufacturing capacity.

## Methods

We used a political economy framework to analyze the effect of varied institutions, political processes, and economic interests on public policy outcomes. We assumed that institutions are sources of predictability and credibility. Through this framework, we examined how political and/or economic interests can impede or facilitate CAMR from achieving its objective.

We conducted qualitative interviews and analysis to assess stakeholder perceptions and attitudes to CAMR. We analyzed our data according to stakeholder group and coded for major themes guided by the research objectives and interview questions [8]. NVivo 7.0, a qualitative analysis software program, was used to analyze the interview data. We estimated a sample size of 4–6 people for each group, based upon a similar study [9]; however we interviewed with the goal of reaching theme saturation.

In total, we conducted 19 open-ended interviews with stakeholders from different interest groups, including 4 health-policy-makers from developing countries, 5 officials from the Canadian Government, 2 representatives from the Canadian generic pharmaceutical industry, 2 representatives from the research-based pharmaceutical industry, 3 representatives from non-governmental organizations, and 2 from international organizations. Interviews were conducted between July 2006 and October 2007, were conducted either face-to-face or over the telephone, and ranged from 30 minutes to one hour in length. Ethical approval was obtained from the University of Toronto, Research Ethics Board in 2006. Informed consent was obtained from all participants prior to the interview. With the permission of the study participant, interviews were tape-recorded and transcribed verbatim. Selected checks on the accuracy of transcripts were conducted prior to analysis.

To assess the stakeholders' positions, we explored their views on intellectual property protection and its role in global drug access. As well, stakeholders were asked to describe their participation during the creation of CAMR, and whether their interests were addressed.

## Results

### Developing countries

We found some common themes in our interviews regarding the needs of developing countries; even though we make the point that every country's situation is unique. An underlying theme throughout the interviews was the need to understand and appreciate the circumstances and systems in beneficiary countries. For example, the developing country representative that was most familiar with CAMR, found it an overly complex and unusable legislation. The lack of direct input into the legislative process from developing country governments, who are the intended beneficiaries of the legislation, was viewed as problematic. As one developing country representative notes:

"...I think that our view is simple: that the needs of developing countries must be taken into consideration any time international programs have been set up, because the beneficiaries of the programs must be able to be part of the planning process. And therefore, if you're trying to increase access to medicines and then you set up a new criteria and processes, that in themselves becomes the barriers, and then we have not done much"

Administratively, the CAMR assumes that developing country governments have the requisite knowledge and human resource capacity to make use of the regime. One respondent emphasized the need for technical support to better facilitate its use, however it must be tailored to the specific needs of the country in question. Developing country representatives are open to using CAMR to import medicines, however their responses suggested that it doesn't address their needs and that the incentives are not there to warrant using the system.

"...Bill C-9 is limited to certain specific medicines that some of them we do not really need. So, once you issue a compulsory license, you also want to have the one that gives you the maximum returns. I mean cheaper in cost, and less administrative issues that surround the problem..."

CAMR's limited list of drugs, modeled after the WHO Essential Medicines List, appears to be a disincentive for developing country beneficiaries as it includes many drugs that are already available off-patent elsewhere. Developing countries are interested in affordable second and third-line antiretroviral therapy and antimalarial agents, many of which may not currently be on the list. Others suggested that drug needs vary depending on patent regimes in place, epidemiological profiles of a given country and budget capacity.

"We also have to keep in mind that, when a population has a higher life expectancy rate, chronic degenerative diseases will increase, and this will bring along different treatment needs. Generally, we're visualizing cancer will be on the rise for this reason and we'll probably be needing more cancer drugs...drugs to treat other degenerative diseases, diabetes, respiratory illnesses, which need new molecules to bring any benefits."

In our interviews, the developing country representatives framed access not only as an issue of affordability, but also one which demands consideration of sustainability of treatment and supply. One policy-maker provided examples of stock-outs in his country leading to interruption of treatment and subsequent increased drug-resistance. He emphasized the challenges of ensuring continuity of treatment in resource-limited countries that cannot secure sustainable, affordable, quality pharmaceuticals. One respondent viewed the development of their local industry as a means of achieving sustainable drug supply.

"...take for instance in my country, we also think that you don't only give us fish because we want fish, but you teach us how to do the fishing. Because as a country we must begin to think ten or fifteen years to count, what would happen if we just continue to just import from developed countries?"

### Pharmaceutical industry

#### Generic industry

A theme emphasized by the generic drug industry in Canada is that the legislation must provide better commercial incentives in order to meet its policy objectives. The "Good Faith Clause" requires that the average price of the licensed drug be less than 25% of the equivalent patented brand name drug in Canada, otherwise the generic company will be subject to litigation from the patent holder. Companies viewed these provisions as subjecting them to considerable liability for little economic benefit.

"...yes, they (generic drug companies) have some corporate responsibility, obviously, they're drug companies with CSR, but passing legislation the way the government did, which is intended to be non commercial, and then saying they did wonderful things, again, it's not realistic. You have international companies, who operate in Canada and say, why should I go to Canada and lose money because the Canadian government has an objective."

Respondents acknowledged many other disincentives. First, one disincentive is the cost associated with making use of the legislation. Generic drug companies are required to negotiate a voluntary license with potentially multiple patent holders pursuant to this law. Respondents described this as a lengthy, complex and expensive process, with no time limit given to these requirements. Second, it does not provide sufficient economic compensation to generic drug companies so they will pursue the legally costly compulsory license process. Third, in the event that a company is committed enough to go forward with a compulsory license, it is limited to only two years, subject to a one-year renewal, and the quantity of the license is limited to that which was originally applied for by the country. Given the heavy front-end investment demanded from generic companies, these limits do not provide any prospect for a large or long-term market and give these companies little incentive to engage in this legislation. This is particularly the case if a company would need to adjust and/or increase their manufacturing infrastructure for products which are not normally part of their product portfolio.

"Well, we might end up with a couple of orders, but at the end of the day we won't make any money out of it, and I'm going to get to a point where someone else comes along, like [NGO], and say "we want this other compound", I'm not going to be able to develop it, because I'm in business to make money and I can only do so many products."

A major drawback acknowledged was the lack of accounting for global market realities. Respondents acknowledged that the vast majority of cheap, generic drugs are being exported to least developing countries from countries with such as India, and Brazil. From a price-point, Canadian producers are unable to compete given input costs are significantly lower in these countries.

"My average production per person is $15 [in Canada] and in India is under $1 an hour. The facilities that we've put up in Canada; we've just put up $1 billion into all these facilities to make the 20 million dosages...I could have put that up in India for less than $130 million. That's almost 10-fold the cost!"

Generic companies expressed their disappointment that the Canadian government did not seek better counsel from them in order to make this legislation work.

"...we've asked during the phase for the Canadian government actually partner with some of our companies that were interested, and come on board, work with us, take us through the system and then actually help us, partner with us, either in terms of guaranteeing a certain purchase, a certain quantity and price for the product or helping us negotiate with the international agencies and/or companies to help the generic find partners. It's kind of a new business venture they're asking companies to get into, but the Canadian government has been very unwilling to do that. They've said no, we've passed the legislation, now you have to figure it out."

Overall, respondents recognized that the impact of the TRIPS Agreement on the generic pharmaceutical industry, and in particular the Indian generic industry, would be much more pronounced in the near future. In this sense, they see the importance of the Paragraph 6 Decision and CAMR. However, most respondents saw the TRIPS Agreement and the Paragraph 6 Decision themselves as the fundamental frameworks requiring reform. Generally, generic companies are discouraged about the prospects of making this Paragraph 6 mechanism work.

#### Research-based industry

One of the key informants from the research-based pharmaceutical company expressed support for the intent of the legislation. This is congruent with the Canada's Research-Based Pharmaceutical Companies' (Rx&D) public statements. He recognized the lack of success of the regime and that this was problematic, however he emphasized that he is not raising specific concerns with the regime. He viewed the industry's involvement with CAMR as an opportunity to provide input and help shape the initiative towards the ultimate goal of achieving drug access. The representative emphasized a need to achieve a balance between encouraging innovation and improving access.

"We supported the WTO decision, and publicly continue to support it. So, did we want it to be the best potential mechanism possible? Yes. Did we have some potential concerns of what could happen and what would actually be fruitful in terms of what's needed to do? Yes, I think we raised a few of those questions. But the ultimate position was of support, even publicly, even until recently during the AIDS Conference. It's also fair to say there's improving access, and there's the need to balance that with encouraging innovation. And I think that whenever you're in a discussion about this type of issue, we're looking for that balance. But the fact that the industry has publicly supported the initiative highlights overall the position overtime."

The representative viewed the innovative industry's role as a leader in improving drug access through drug and vaccine discovery and development, working in partnerships and helping build infrastructure. He emphasized the view that patents in many of these developing countries are not an issue, and that the problem of access is multi-faceted, with the lack of infrastructure as being critical. Still, he acknowledged the lack of consensus on these issues and suggested that the solution is to get all stakeholders moving towards the same goal.

"But I think that part of the challenge is to get everybody moving in the same direction, the innovative sector, the brand and generic sector. We also need the support of the AIDS groups and the country in question. I think we need a leadership role to be played there, and, given the divergent interests and the people involved... I think that people's intents are good, but perhaps there needs to be an objective, detached party to move them forward."

On the other hand, the second informant from the research-based pharmaceutical industry expressed no support for CAMR, considering it based upon the false premise that pricing is the main barrier for drug access. According to this representative, the global drug market debate has solved the issue of high priced drugs, and is now focusing on achieving a sustainable drug supply chain.

"The price issue is off the table... We've recognized that part of the problem of access to medicines is resources, health care capacities, infrastructure and access to sustainable financing. We need to find one certain model of efficiency on the ground into how to actually distribute the medicines... [such as] setting up a network, a flow chart of health care."

This representative viewed CAMR as the result of a political agenda, as opposed to addressing the main issue of enhancing equitable drug access. Canadian generic companies have unrealistic expectations from CAMR and they have no experience with the complex realities of developing country markets.

"I'm not blaming the government at the time...Politics sometimes trumps policy, and this is a great example of politics, pure politics trumping sensible and sound policy."

The informant emphasized that Canada should instead focus on delivering organized, well-informed programs that would match the 0.7% Gross Domestic Product destined to foreign aid previously promised.

### International organizations

There was clear lack of confidence regarding the potential impact of this legislation from both representatives of international aid organizations.

Although the first informant conceded that the legislation and the larger WTO Paragraph Six decision were important steps towards enhancing global access to medicines, she believed that the CAMR will have limited impact.

"...it's a really narrow slice of the piece....being a narrow slice, I meant more than in terms of Canada, we've done this, and we've done a great thing. They've done a great thing to help a few countries, but it's not such a huge step."

"The value of the Canadian legislation is only as good and strong and useful as what each country has in terms of its own legislation and as much as they feel empowered to take advantage of those compulsory licensing arrangements. And not a lot of countries do; you have to be a pretty strong country to do it."

Intellectual property protection was cited as necessary for companies to make advances in new products, such as AIDS and cancer drugs, but she said that countries focus first on access to drugs on the essential medicines list, which are largely off-patent. The issue of access is multi-faceted and many of the barriers exist in developing countries themselves: regulatory issues, high taxes/tariffs on imported drugs, manufacturing practices, licensed pharmacists, legislation for market authorization, integrity of the drug supply, and corruption.

"...even if tomorrow all prices are cut in drugs, that's not going to solve the entire problem."

The second representative favored the TRIPS agreement, but recognized that compulsory licensing should be used only as a last resource after all other negotiations with patent holders have failed. Since it reduces incentives for further drug research and innovation, compulsory licensing must be carefully monitored.

According to this informant, CAMR could achieve a significant global impact only if it is implemented in conjunction with remaining problems in drug access, including the need for more efficient drug regulation, professional training and health services improvement. The Canadian legislation does not address these important issues and lacks a general consensus from all parties involved in the process.

### Civil society

Most civil society representatives were in agreement with the intent of the legislation but highly critical of the legislation in its current form. The barriers highlighted by civil society were consistent with those cited by the generic drug industry over the lack of commercial incentive, and concerns of developing countries with respect to their health system issues and broader country contexts. Concerns about the drug list, the voluntary license negotiations, the limited list of medicines and limits on duration and quantity were also emphasized. These were seen as disincentives to both the generic industry and developing countries. Even though Apotex is moving forward with a plan to export drugs to Rwanda, the legislation is still viewed as cumbersome to put in use and not viewed favourably civil society. As one health activist noted:

"It's one drug, for one country and it's taken us from April 2004 to say April 2007, that's three years to get one drug for one country. And it's only a compulsory license to 150000 tablets, which will actually treat 200 people for two years."

The lack of interest until recently by developing countries was attributed to many factors. The process that developing countries must go through to obtain drugs under the CAMR was seen as largely unrealistic.

"Well, it's obvious that nobody's interested. A country that has got a huge death rate from AIDS, they don't have the time or resources to go through this with every single drug a country like Tanzania, you have one person working on international intellectual property."

That no drug is currently available for purchase through CAMR is also seen as a barrier to garnering the interest of potential countries. And the fact that countries must notify the WTO before using the system was seen as a key barrier.

"A country that wants to do this has to stick its neck out, and make an order, and say to the world, and to the Canadian government, 'We intend to use the WTO system, specifically Canada's....' and there will be an immediate backlash when they do this...certainly not before there's a tangible product that they're going to get at the end of the day."

Donor influence on pharmaceutical procurement procedures was also cited. Bilateral donors may attach conditionalities with respect to procurement. Others, such as the World Bank, typically require that countries undertake international competitive bidding for the purchase of pharmaceuticals. The Canadian legislation simply does not fit within those restrictions and does not provide enough incentive to justify breaking these donor arrangements.

"If I'm sitting here, and I'm in Malawi, and I've got $200 million annually from the US for drugs as long as I buy patent drugs, do you think I'm going to thumb my nose at that? It's part of the bigger architecture."

Related to this is the perception of an inactive government. Most civil society groups stated that the government needs to do much more on both the supply and the demand sides. This involvement includes working with developing country bureaucrats and officials, providing technical assistance, and working with donors in those countries to ensure that the regime works within the donor requirements.

"Those that don't have the manufacturing capacity often don't have the negotiating capacity with strong donor groups. Therefore government of Canada needs to do some strong advocacy and work with the rest of the donor group to help the country use that."

### Canadian federal government

Overall, bureaucrats were reluctant to label the CAMR as either effective or not. They emphasized the difficulty of creating legislation true to the WTO Doha Accord without having a precedent to follow. Generally, bureaucrats felt that the CAMR was an important policy to give effect to the global trade mechanism and encourage other countries to follow suit. However, they recognized that the policy was wanting in parts and deferred to the review process as an effective mechanism to evaluate the problems and amend accordingly.

One bureaucrat speculated that the lack of use of the CAMR was largely a problem on the developing country side. Countries show interest in the legislation but they are not notifying the Canadian Commissioner of Patents of their intent to use the legislation. While no definitive reasons were given, he speculated as to whether there was a lack of clarity on WTO or Canadian notification, pressure from stable pharmaceutical providers not to notify, or a disconnect between CAMR and a country's economic goals.

"A lot of their industrial goals are not to have any drugs shipped from Canada, but to build their domestic capacity. So a lot of them brought up that issue; the waiver is interesting, but our primary concern is to build a supply in our own country."

Moreover, the problems are seen as less of an issue with the CAMR as they are a function of the international architecture of the WTO Agreement.

"The problem is not as much domestic. They [a generic company] haven't applied to the Commissioner for a license because they need a country to notify them... So the problem is the international machinery, and look, if you're going to change the world and the wait works commercially, you can't do it in a week."

Bureaucrats view themselves as actively disseminating information and engaging countries, companies, and private foundations. But they are aware that the legislation does not provide sufficient commercial incentives for the generic drug industry.

"A lot of the companies are now controlled by international boards, so when the provisions first came through, we got a meeting with 5–6 CEOs of the major generic companies, and we brought them through the process and how you would need to apply. We had a frank discussion about whether they would participate. The prevailing thought was, there was a lot of commercial risk involved, they would very likely not be able to convince their boards to embark on that kind of thing, so there was very little interest because it's commercially risky, there's really not much point for them."

An opposition party politician assumed a very critical position, seeing the legislation as a policy failure.

"I don't want to have this legislation as a political shield that says we're contributing to humanity in developing nations when they can't really make use, or the bill doesn't provide the remedy at the end of the day. ....If it stays the current way and we can't get any drugs to anybody, then I think it's one of the worst pieces of legislation ever passed in Parliament. It just allows us to say we're so good and so humanitarian, and by the way, you really can't use it."

A majority party politician remained more impartial on the success of the CAMR, acknowledging that if it wasn't working, something had to be done. She emphasized the importance of protecting intellectual property, shareholder investments and encouraging innovation.

"At the same time, you have to also protect those patents, because you have people, ordinary people, ordinary Canadians who are not wealthy that may invest $1000 or whatever, and hold one or two shares in a variety of companies, including maybe pharmaceutical-based, research-based pharmaceuticals. They have a right to have their investment protected, and try to make some money off of it. It's perfectly justifiable. So the companies have an obligation to their shareholders, because those are the ones who provided the capital that they then used to actually do the research...."

Yet, the politician clearly stated that if the bill isn't working, it should be reassessed and amended to be more effective.

## Discussion

CAMR seeks to reconcile both commercial and humanitarian goals but it has yet to succeed on both accounts. For the primary users of the legislation – developing country representatives and the generic drug industry – CAMR fails to adequately address their needs. The legislation needs to include commercial incentives to galvanize the generic drug industry to make use of this legislation. While Apotex has demonstrated good will by agreeing to manufacture medicines for Rwanda, it remains to be seen if this is a one off event or if this will be a more long-lasting arrangement. If, and only if, it is the latter, then the legislation may be of value towards ensuring sustainable pharmaceutical supply.

From the perspective of developing country representatives, the intended beneficiaries of this legislation, CAMR is almost inaccessible. To be sure, the complexity of the regime may be attributed in part to the TRIPS Agreement. Some interviewees acknowledged that international trade agreements themselves, which CAMR is situated within, are responsible for barriers. Still, critics agree that these international mechanisms must be simplified to better enable low to middle income countries to use compulsory licensing and other TRIPS flexibilities [4]. Given these circumstances, the Canadian legislation took a complicated regime and, based upon our findings, made it even more complex.

The CAMR works on the assumption that developing country governments have the requisite knowledge and human resource capacity to make use of the regime seamlessly. In crisis situations, government officials will not opt to deal with cumbersome administration in order to get drugs to those in need. They will seek expeditious and simple solutions to stop people from dying or being sick from lack of access to medicines. Furthermore, the legislation does not offer sufficient incentives for countries to turn to Canada for help when needed drugs may be procured cheaply from another source country, such as India, which has very competitive prices and has also amended its patent law to act as a source country for countries wanting to make use of the Doha Declaration on TRIPS and Public Health.

In the long term, developing and least developing countries seek sustainable solutions to meet the health needs of their population. Many despite the economic costs want to build up their own capacity to produce medicines and develop their local industry so that drug supply can be assured nationally and there is no dependence on external supply. This requires however the requisite technology transfer which has been limited at best pursuant to the TRIPS Agreement. Measures such as CAMR and the Paragraph 6 Decision are intended to be stop gap measures – policy instruments to address urgent and present needs that are otherwise unfulfilled. A balance, however elusive, needs to be struck between trying to address the access crisis in a timely manner while developing the necessary foundations for sustainable access and treatment. Governments, like Canada, can fill this role by offering assistance to relevant sectors including trade and industry to increase their technical capacity, which is in fact required by the TRIPS agreement.

International development to succeed must have local input and be mindful of variation in country needs. This is an approach, which has long been advocated by development agencies. Still, this legislation appears to be out-dated and out of synch by imposing a"one-size" fits all approach to drug access. We found that there is a common perception that the legislation has not reflected the health and economic priorities of developing countries. There needs to be sufficient counsel from developing countries themselves and they need to be involved in the planning process to ensure the content and the process of the legislation works seamlessly in their favour. Canada's intention to correct an unfair trade regime, one that was clearly not in the interest of developing countries, is at a cursory level positive, but unless realistic and appropriate incentives are in place, the legislation will simply be an international embarrassment.

The brand name industry's perception, as reflected by our results, that high prices are not the main barrier to drug access is indicative of their position on CAMR and commiserate with global policy debates. While this study illustrates that there are many key components to ensuring drug access in developing countries, results also showed that the affordability of pharmaceuticals remains a problem. Industry's strong resistance towards allowing ample flexibility in intellectual property law to achieve public health objectives has been clearly shown in the past. Given these entrenched interests, meaningful policy that aims to reconcile intellectual property protection and access to medicines cannot be realistically founded upon consensus between all stakeholders. Effective policy requires leadership and courage on behalf of the government to make amendments and provisions necessary to create the market conditions necessary to stimulate the flow of drugs into these countries. This will take creating policy that prioritizes humanitarian over commercial goals.

Results should be considered in the context of the study's limitations. First, our study focused on perceptions of CAMR but did not evaluate the application of CAMR directly. An evaluation of the current Apotex-Rwanda case would be ideal to examine the technical problems of the legislation; however this was not our objective. We aimed to capture a multiple stakeholder critique of CAMR's overall goals as well as its policy design, by incorporating broad, global perspectives on drug access along with those more intimately familiar with the specifics of CAMR. Second, we interviewed with the goal of theme saturation, however our sample size was limited by recruitment and project constraints. We aimed to achieve a diversity of views across the five stakeholder groups, which we believe represent a multiple-faceted view of the issues at hand.

## Conclusion

Based on our findings, CAMR appears to be more powerful symbolically than in practice. The planned export of generic antiretroviral medicines to Rwanda suggests that the legislation may prove to be more workable than originally anticipated. It is still too early to determine this and this case needs to be examined closely in order to draw a more conclusive view. Nonetheless, we still contend that to meet its intended international health objectives, this legislation needs to be better informed of developing country needs and global pharmaceutical market imperatives. It must be simple to implement and address the vital issue of drug sustainability. This would likely include components related to facilitating local production and technology transfer, and of course, specific country needs. Finally, we contend that serious public policy change cannot strike a balance between all vested interests. It is almost impossible to derive fundamental policy change that will satisfy all vested groups. Here, CAMR must remain faithful to the Doha Accord and ensure that public health is kept as the priority.

## List of abbreviations

AIDS Acquired Immune Deficiency Syndrome

CAMR Canada's Access to Medicines Regime

CSR Corporate Social Responsibility

NGO Non-governmental organization

PEPFAR U.S. President's Emergency Plan for AIDS Relief

Rx&D Canada's Research-Based Pharmaceutical Companies

TRIPS Trade-Related Aspects of Intellectual Property Rights

WTO World Trade Organization

## Competing interests

The author(s) declare that they have no competing interests.

## Authors' contributions

JK designed the study, conceptualized and drafted the paper, and participated in data collection and analysis. LE contributed to study design, drafting and revising the paper and participated in data collection and analysis. APC contributed to drafting and revising the paper and carried out data collection and analysis.
